# Materials Prediction via Classification Learning

**DOI:** 10.1038/srep13285

**Published:** 2015-08-25

**Authors:** Prasanna V. Balachandran, James Theiler, James M. Rondinelli, Turab Lookman

**Affiliations:** 1 Theoretical Division, Los Alamos National Laboratory, Los Alamos, NM 87545, USA; 2 Intelligence and Space Research, Los Alamos National Laboratory, Los Alamos, NM 87545, USA; 3 Department of Materials Science and Engineering, Northwestern University, Evanston, IL 60208, USA

## Abstract

In the paradigm of materials informatics for accelerated materials discovery, the choice of feature set (*i.e.* attributes that capture aspects of structure, chemistry and/or bonding) is critical. Ideally, the feature sets should provide a simple physical basis for extracting major structural and chemical trends and furthermore, enable rapid predictions of new material chemistries. Orbital radii calculated from model pseudopotential fits to spectroscopic data are potential candidates to satisfy these conditions. Although these radii (and their linear combinations) have been utilized in the past, their functional forms are largely justified with heuristic arguments. Here we show that machine learning methods naturally uncover the functional forms that mimic most frequently used features in the literature, thereby providing a mathematical basis for feature set construction without *a priori* assumptions. We apply these principles to study two broad materials classes: (i) wide band gap AB compounds and (ii) rare earth-main group RM intermetallics. The AB compounds serve as a prototypical example to demonstrate our approach, whereas the RM intermetallics show how these concepts can be used to rapidly design new ductile materials. Our predictive models indicate that ScCo, ScIr, and YCd should be ductile, whereas each was previously proposed to be brittle.

The advent of pseudopotential theory for solids marked a key period in the computation and application of orbital radii concepts. Simon and Bloch first utilized model potentials in pseudopotential calculations and showed their relevance in reproducing properties of solids that depend explicitly on valence energy, such as ionization energies, equilibrium geometries, force constants, and dissociation energies^1^,^2^. They defined orbital radii by finding the classical turning points or central balance points from a model nonlocal hard-core pseudopotential [*V*(*r*)], where the sum of the repulsive centrifugal and “Pauli forces” exactly cancel out the attractive Coulombic force exerted by the nucleus^3^,^4^. The nonlocal nature of the pseudopotential gives an angular momentum (*l*) dependence, yielding a radius (in units of angstroms) at *V*(*r*) = 0 for the valence electrons in *s*-, *p*-, *d*-, and *f*-like orbitals for an atom or a free ion. There are different forms of *V*(*r*) and their exact derivations are well documented in the literature^1^,^2^,^5^,^6^,^7^.

Our interest in orbital radii for materials informatics, *i.e.*, the growing field focused on using information science methods to understand condensed matter systems, is motivated by several reasons. First, orbital radii are based on model pseudopotential [*V*(*r*)] fits to spectroscopic data, which gives a straightforward reference frame for extracting relative chemical and structural trends^8^. Second, they are transferable from one compound to another. Third, for a given atom (or an ion), depending on its electronic configuration, radii for *s*-, *p*-, *d*-, or *f*-like orbitals exist unlike the empirical scales^9^,^10^ where atomic (or ionic) radii are a single-valued quantity. As a result, this relatively simple physical basis provides more flexibility in exploring and understanding electronic and/or atomic structure—property relationships in complex materials. For example, the orbital radii are able to capture the distance to maximum radial charge density, which determines the interatomic distances and angles in crystals^5^,^9^.

Although the orbital radii are not directly measured from experiments and unsuitable for full electronic structure calculations, it has been hypothesized that linear combinations of localized *s*-, *p*-, *d*-, and *f*-like orbital radii (or their reciprocal) give a qualitative measure of bond covalency and orbital electronegativity^5^,^8^. The underlying rationale for considering linear combinations of orbital radii is rooted deeply in the valence bond theory of solids proposed by Pauling^11^, which provides a conceptual framework for discussing crystal structures and their energetic trends in terms of overlap or hybridization of atomic orbitals^3^. Burdett and Price also used the extended H
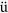
ckel theory to argue the linkage between electronic energies and linear combinations of reciprocal orbital radii that capture the orbital interactions between *ss, pp, sp*, and *ps* contributions to the stabilization energy^12^.

Bloch and Simon were among the earliest proponents of utilizing orbital radii for studying structural trends in solids^3^. They demonstrated that in *s*-*p* bonded elemental solids, the fractional difference between the maxima of *s*- and *p*-radial functions could serve as an “index” (*i.e.* feature) that separates covalent, fcc, hcp, and bcc structures^3^. Later, St. John and Bloch extended the approach to classify crystal structures of wide band gap octet AB compounds^4^, following the original work of Mooser and Pearson^13^ that uses average principal quantum numbers and Pauling scale electronegativity differences^14^. One of the key contributions from St. John and Bloch is the suggestion of a *functional form* for linearly combining the *s*- and *p*-like orbital character of the radii, which led to the nomenclature^5^ of *r*_*σ*_ and *r*_*π*_ as the two principal feature sets for AB compound classification:
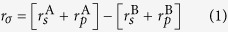

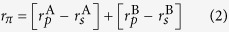


While *r*_*σ*_ (equation 1) captures the electronegativity difference between A- and B-atoms, the feature *r*_*π*_ (equation 2) was identified to represent the *directional* nature of bonding through hybridization between *s*- and *p*-orbitals of A- and B-atoms. Since then Phillips^15^,^16^,^17^, Chelikowsky^5^, Littlewood^18^, Zunger^19^,^20^, Cohen^21^, Andreoni^22^, Burdett^12^, Rabe^23^ and others^24^,^25^,^26^,^27^ have independently applied these orbital radii concepts, including the *r*_*σ*_ and *r*_*π*_ form without *post-facto* reformulation, to classify structures and properties of many other binary and multicomponent crystalline compounds with varying degrees of accuracy and success. As the understanding evolved, modifications to the original form of *r*_*σ*_ and *r*_*π*_ were suggested, largely based on intuition from domain knowledge or physical understanding of the known materials.

Without such understanding, however, the successful formulation of predictive theories for new compounds becomes a non-trivial endeavor. We therefore pose the question: *Can one improve the predictive capabilities of the orbital radii by uncovering the natural form in which they should be combined?* Plainly, is there a simple and tractable mathematical formalism that allows one to automatically construct linear combinations of orbital radii from data, irrespective of the chemical and/or structural complexity of the material? Are there other linear combinations of orbital radii, in addition to the *r*_*σ*_ and *r*_*π*_, for AB compounds? We are not the first to raise these questions^12^,^22^; there are many debates in the literature about the validity of *r*_*σ*_ and *r*_*π*_, and suggestions to explore new functional forms that complement *r*_*σ*_ and *r*_*π*_^22^.

In this work, we use *machine learning* (ML) methods to show that linear combinations of orbital radii can be constructed directly from the data without requiring domain knowledge of a materials class. The wide band gap AB compounds form an ideal starting point to address our main question, because the structures of these materials are well-characterized. We show in the AB family that the functional form of *r*_*σ*_ and *r*_*π*_ can be reproduced solely from data using ML, and furthermore, new linear combinations are uncovered which give more versatility to the orbital radii approach for extracting structure-property relationships. Next, we apply the method to the more complex RM intermetallics (R and M are rare-earth and main group or transition metal element, respectively), where at present *r*_*σ*_ and *r*_*π*_ are poorly defined or do not exist. We classify their mechanical properties into two groups, namely ductile and brittle, and uncover orbital radii-based ‘selection-rules’ that give excellent agreement with computationally intensive density functional theory (DFT) calculations. We demonstrate the predictive power of our methods by identifying new and yet to be synthesized ductile RM intermetallic compounds: We predict ScIr to be ductile, although it was originally proposed as brittle in the literature. Results from ML, electronic structure calculations, and Zener anisotropy ratio data support this prediction. Furthermore, we predict using ML that ScCo and YCd are potential ductile materials albeit our electronic structure calculations and Zener anisotropy ratio data are not as conclusive. These results have major implications beyond searching for materials with improved mechanical properties; the unbiased formulation of orbital radii for classification and property prediction can be readily applied to the electronic, magnetic, and optical function of more complex materials where only limited models of the properties are available, enabling an expansion of new materials for experimentation.

## Results

### Data sets

#### AB *compounds*

**Figure 1:**
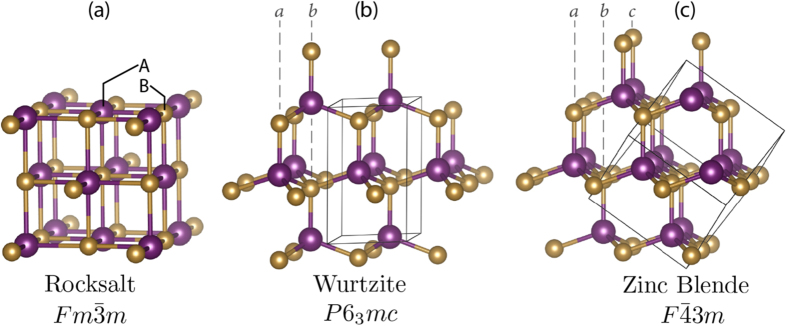
Crystal structures of AB solids considered in this work. (**a**) Rock salt structure in space group 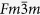, (**b**) Wurtzite structure in space group *P*6_3_*mc*, and (**c**) Zinc blende structure in space group 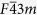. The positions of A (in color purple) and B (in color gold) atoms are labeled in the figure. Solid lines indicate the unit cell.

The data set for this work is made of 55 AB compounds taken from the recent work of Saad *et al.*^25^. Each AB compound is labeled uniquely in one of three crystal structure forms: rocksalt (R), wurtzite (W), or zinc blende (Z) as shown in Fig. 1. There were a total of 28, 8, and 19 AB compounds in R-, W-, and Z-structures, respectively. The R-structure has a 6-fold nearest neighbor coordination environment, whereas W- and Z-structures have 4-fold coordination. The stacking sequence and bond distortions in the AB_4_ tetrahedra distinguish the W- from Z-structures. For instance, in W- and Z-structures we observe a stacking sequence of —*abab*— (Fig. 1b) and —*abcabc*— (Fig. 1c), respectively. The AB_4_ tetrahedron in the Z-structure are ideal, *i.e.*, each has four equidistant A–B bond lengths, whereas in W-structure only three of the four A—B bonds are of equal length owing to the polar symmetry. Note that in Saad *et al.*, six (out of 55) AB compounds were originally labeled as dual structures, where the ground state structures were designated as borderline due to close energetic competition. We performed DFT calculations on those six compounds and re-labeled them uniquely to a single structure-type based on the phase with the lowest energy (amongst the three R, W, and Z-structures). See Supplementary Note 1 and Supplementary Table 1 for the total energies.

We use two sets of orbital radii scales for the feature set: Chelikowsky (C)^25^, and Waber-Cromer (WC)^28^. Orbital radii from the Chelikowsky scale are defined by classical turning points [*V*(*r*_*l*_) = 0] of hard core nonlocal pseudopotentials total energies from DFT in the local-density approximation (LDA). The Waber and Cromer scale, on the other hand, uses the self-consistent Dirac-Slater eigenfunctions and the orbital radii correspond to the principal maxima in the charge-density distribution function for a specific orbital of an atom. This orbital radii scale is for neutral elements.

In the Chelikowsky scale, the *p*-orbital radii (*r*_*p*_) for an atom is always greater than that of the *s*-orbital radii (*r*_*s*_), however, this it is not the case in the Waber-Cromer scale. For instance, consider the Cd atom: Waber-Cromer tabulated the values of 4*p*, 4*d*, and 5*s* orbital radii as 0.445, 0.505 and 1.18 Å, respectively; in this formalism, *r*_4*p*_ < *r*_5*s*_. Howeve*r*, in the Chelikowsky scale *r*_*s*_ and *r*_*p*_ fo*r* the Cd atom are 0.67 and 1.26 Å, respectively; here, *r*_*p*_ > *r*_*s*_. In the case of Si atom, Waber-Cromer scale lists the 3*s* and 3*p* orbital radii as 0.904 and 1.068 Å, respectively and the Chelikowsky scale has *r*_*s*_ and *r*_*p*_ as 0.66 and 0.88 Å, respectively. We maintain consistency between the two scales in our data set by tabulating the smaller orbital radii first, followed by the larger one (irrespective of their principal quantum number). In both scales, the pseudopotential model, [*V*(*r*)], replicates only the valence electronic states.

We also follow the conventional notation of *r*_*s*_ and *r*_*p*_ for the Chelikowsy scale, whereas we revise our notation to *r*_*i*_ and *r*_*o*_ for the Waber-Cromer scale; *r*_*i*_ and *r*_*o*_ stand for radius of inner and outermost orbitals, respectively. We only consider the radii of *s*- and *p*-orbitals and neglect the *d*-orbital in both scales. Although the Chelikowsky’s scale has been used before for classifying the crystal structures of AB compounds^25^, this is the first time that the Waber-Cromer’s scale is explored for these purposes. One of the main advantages of the Waber-Cromer scale is that the orbital radii data have been tabulated for the majority of elements in the periodic table—including lanthanides and actinides.

#### RM *intermetallic*s

**Figure 2:**
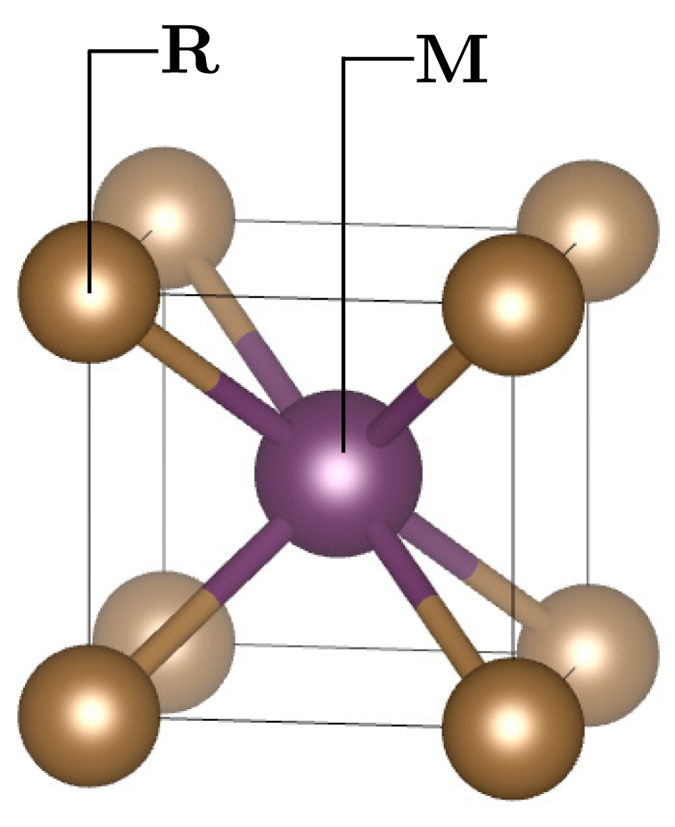
Cubic B2 CsCl crystal structure-type of RM intermetallics in space group 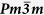. The positions of R (in color gold) and M (in color purple) atoms in the crystal structure are labeled in the figure. There are two atoms per unit cell. Solid lines indicate the unit cell.

Our training data set for classification learning comprises 30 RM compounds, with mechanical properties experimentally measured through tensile and impact tests (19 are ductile and 11 are brittle)^29^. We construct a data set for ML using the same label as reported by Gschneidner *et al.*^29^ and characterize each RM compound using only the Waber-Cromer orbital radii scale. We were are unable to explore the Chelikowsky scale for this problem because it does not contain radii for the rare-earth elements. For the R-atom, we used the *s*-, *p*-, *d*-, and *f*-orbital radii; whereas for the M-atom we used *s*-, *p*-, and *d*-orbital radii. We also constructed an additional *virtual* or *unexplored* set of 113 RM compounds. Our objective is to build a classification model on the training set and, in turn, use it to predict the mechanical properties (ductile or brittle) of unexplored 113 compounds. Note that all 143 compounds are assumed to be fully stoichiometric and to have the cubic **B2** CsCl crystal structure-type (see Fig. 2).

### Crystal Structure Classification of Wide Band Gap AB compounds

**Figure 3:**
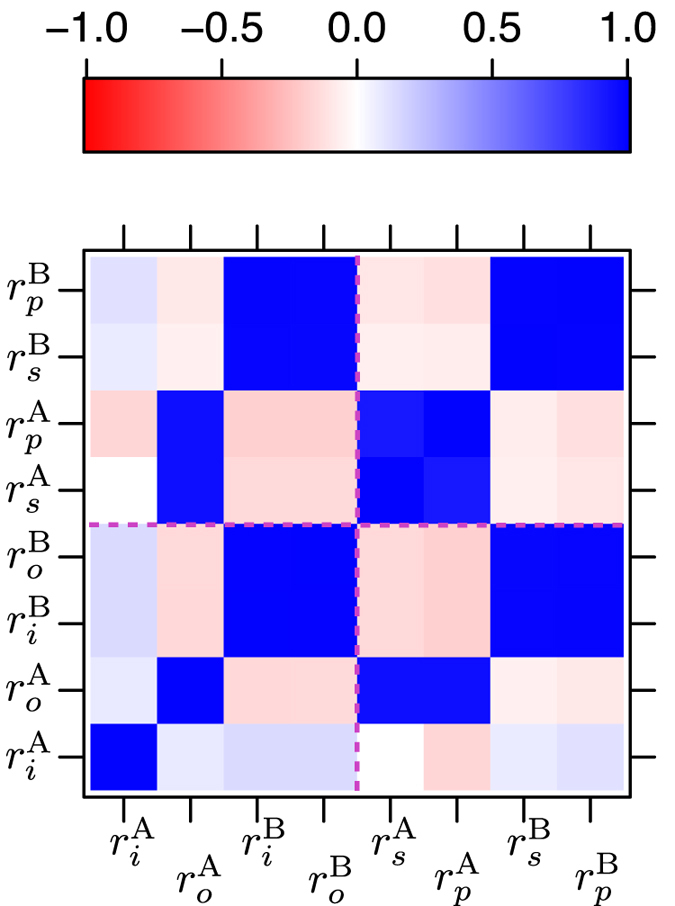
Statistical correlation map for the two orbital radii scales. *r*_*i*_ and *r*_*o*_ are the inner and outermost orbital radii, respectively, of A- and B-atoms from the Waber-Cromer scale. *r*_*s*_ and *r*_*p*_ are the *s*- and *p*-orbital radii, respectively, of A- and B-atoms from the Chelikowsky scale. Red (negative) and blue (positive) color indicate strong inverse and direct correlation, respectively. Pink dotted lines are drawn as a guide to the eye for improved readability, which separates the Waber-Cromer scale from Chelikowsky scale.

In Fig. 3, the bi-variate statistical correlation map between the *s*-(*i*-) and *p*-(*o*-) orbital radii of A- and B-atoms for the two scales are shown. Negative and positive signs indicate inverse and direct correlation, respectively. Figure 3 uncovers key similarities and differences between the two scales. In the Chelikowsky scale, *s*- and *p*-orbital radii for both A- and B-atoms correlate strongly. On the other hand, in the Waber-Cromer scale only 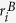 and 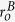 for the B-atoms show strong correlation. In the case of the A-atom, we find that only 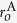 correlates strongly with both 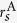 and 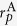. This is one of the important differences between the Waber-Cromer and Chelikowsky scales.

**Figure 4:**
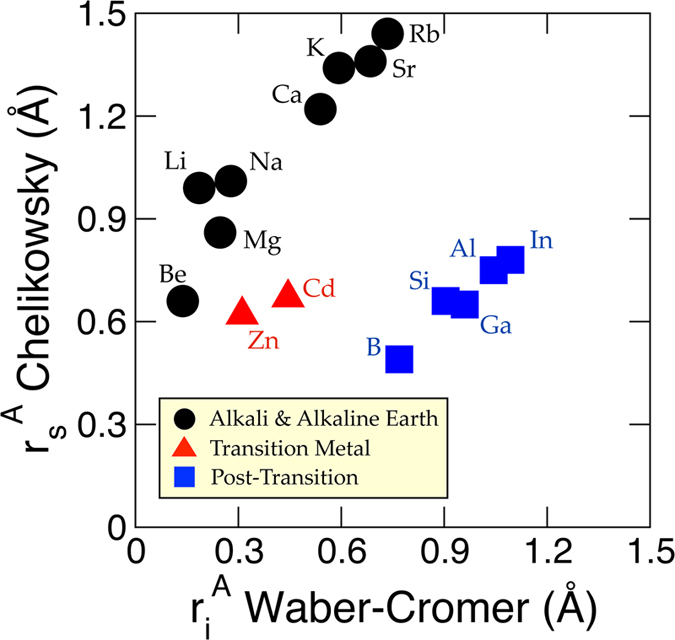
Difference between Waber-Cromer (abscissa) and Chelikowsky (ordinate) scales. 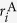
 and 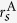 are the inner and *s*-orbital radii of Waber-Cromer and Chelikowsky scales, respectively.

A scatter plot between 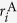 (from Waber-Cromer scale) and 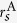 (from Chelikowsky scale) is shown in Fig. 4, where the underlying periodic trends are apparent (identified based on prior knowledge about these elements): (i) Be, Li, Na, K, Rb, Mg, Ca and Sr (alkali and alkaline earth elements) (ii) Zn and Cd (transition series elements), and (iii) B, Si, Ga, Al and In (post-transition series elements). We observe piecewise linear relationships within each elemental series, but collectively the correlation coefficient is small. As noted before, the orbital radii from the Chelikowsky scale has its origin in nonlocal pseudopotentials from DFT within the LDA, while the Waber-Cromer scale is the principal maxima in the charge density from the Dirac-Slater wavefunction model. We attribute the absence of correlation between 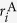 and 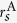 to the two different *V*(*r*) pseudopotential models.

After correlation analysis, we constructed two separate data sets (each dataset is now a 54 × 4 matrix), one for each orbital radii scale. Each data set was then subjected to principal component analysis (PCA), where we first autoscaled the data (*i.e.* each column vector was normalized to have zero mean and unit variance) and calculated the sample covariance matrix (Σ). Eigenvalue decomposition of the Σ-matrix produces two new matrices: eigenvalues and eigenvectors. Since we have a 54 × 4 data matrix, the eigenvalue decomposition procedure produces four eigenvalues (in the form of a diagonal matrix) and four eigenvectors. Each eigenvector [also referred to as principal component (PC) loading] is a *linear combination* of the weighted contribution of the *r*_*s*_ (*r*_*i*_) and *r*_*p*_ (*r*_*o*_) orbital radii of A- and B-atoms, and the eigenvalues indicate the % variance captured by the corresponding eigenvectors. We then project the autoscaled data points onto the loadings, which are called the *PC scores*. Since there are four eigenvectors, we have four PC scores. The difference seen in Fig. 3 between the two scales is manifested in the %-variance data given by the eigenvalues of each PC (Table 1), which suggests that the data structures in both scales are quite different.

A rule of thumb is to plot % variance as a function of number of PC’s and locate the *elbow* in the curve^30^. The elbow generally determines the number of PC’s to be considered for further analysis. In this paper, we consider all PC’s for classification learning.

The functional forms for the *s*- and *p*-orbital radii combinations obtained from the Chelikowsky scale eigenvectors are given in Table 2. Notice the striking similarity in the functional form of C-PC1 and 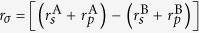. Similarly, C-PC3 and C-PC4 have the functional form of 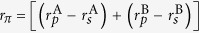, albeit with large difference in the coefficients or weights; however for the given data set, C-PC3 and C-PC4 capture only small amount of variance in the data set (see Table 1).

The C-PC2 is an interesting feature as it captures the orbital radii sum (*r*_*s*_ + *r*_*p*_) of the A- and B-atoms, yet its functional form has hitherto not been explored. Furthermore, it accounts for 44.38% of the variance in the data set (Table 1). Normally, the difference between *r*_*s*_ and *r*_*p*_ is often used to infer the degree of hybridization between *s*- and *p*-orbitals for an atom; the smaller the difference, the greater the potential for *sp*-hybridization and vice versa. However, the functional form of C-PC2 corresponds to an orbital radii sum. We attribute the physical picture of [*r*_*s*_ + *r*_*p*_] to describe the core radius of an atom implying that C-PC2 captures the core radii sum of the atom set (A and B). A scatter plot between *r*_*s*_ + *r*_*p*_ for the A-atom and its corresponding Shannon ionic radius^10^ for the (monovalent and divalent) cations and anions in 6-fold coordination shows a linear relationship (see Supplementary Note 2 and Supplementary Figure 1). As a result, we infer that C-PC2 captures the physics describing the relative close-packed tendencies of various AB compounds.

**Figure 5:**
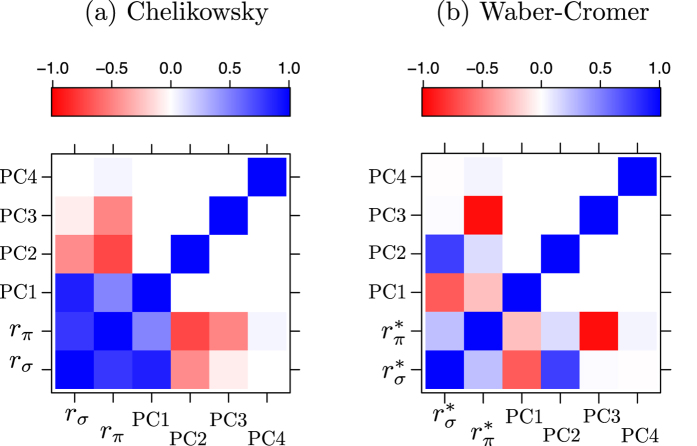
Correlation map between PC scores, *r*_*σ*_, and *r*_*π*_ in (**a**) Chelikowsky scale and (**b**) Waber-Cromer scale. Red (negative) and blue (positive) colors indicate strong inverse and direct correlation, respectively, whereas white color indicates no correlation.

In Fig. 5a, the correlation plot of four PC scores along with the *r*_*σ*_ and *r*_*π*_ features for the Chelikowsky scale is shown. The correlation coefficient (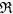) between *r*_*σ*_ and *r*_*π*_ features is found to be 0.63. As expected, we find that *r*_*σ*_ correlates strongly with C-PC1 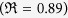; interestingly, *r*_*π*_ is found to correlate with C-PC2 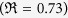. We remark that the sign of the PCs are arbitrary; thus the sign of the correlation (positive, direct or negative, inverse) is not meaningful.

Functional forms for the four eigenvectors from the Waber-Cromer scale are given in Table 2. In terms of trends, as shown in Fig. 5b, we find that WC-PC2 correlates directly with 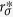 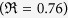, whereas WC-PC3 is found to correlate inversely with 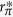 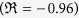. We use an asterisk symbol (*) in *r*_*σ*_ and *r*_*π*_ for the Waber-Cromer scale to differentiate it from that of the Chelikowsky scale. As noted earlier, in the Waber-Cromer scale we use the inner (*r*_*i*_) and outermost (*r*_*o*_) orbital radii, whose principal quantum number can be different. Originally, the *r*_*σ*_ and *r*_*π*_ were conceived for *s*- and *p*-orbital radii that resemble the pseudopotential model of the Chelikowsky scale.

We now classify the crystal structures of AB compounds to one of the three R, W, and Z using the PC scores from both scales and compare them with the canonical *r*_*σ*_ and *r*_*π*_ descriptors. We utilize decision trees and support vector machine (SVM) algorithms for ML (see Methods section). The performance of classifiers were assessed using the full training set and leave-one-out cross validation (LOO-CV), which involves training the ML algorithm on all but one data point and then applying that classifier to the left-out data point. The process is repeated until each compound is left out exactly once and its crystal structure or properties *predicted* from the knowledge of the remaining compounds. LOO-CV is a common procedure used in ML when the training data sets are smaller in size (such as those in this paper). The results from classification learning are given in Table 3. Notice the similarity in the classification accuracies between PCA and [*r*_*σ*_, *r*_*π*_] features in both the scales. The largest difference in terms of accuracy is found to be ~5.5% between [*r*_*σ*_, *r*_*π*_] and PCA in the Chelikowsky scale with SVM. In all other cases, the performances are similar.

In Table 4, we list the most common misclassified AB compounds from the full training set for both scales. We performed DFT calculations (see Methods section) on these five misclassified compounds and the results are also tabulated in Table 4. Except CdSe, the lowest energy structures for the rest of the AB compounds agree well with those of Saud *et al.* We identify CdSe to have the zinc blende (Z) structure from our DFT calculations, although it was originally labeled to be wurtzite (W) by Saud *et al.* Note that the energy difference between Z- and W-structures is as small as 2 meV/formula unit (f.u). The 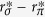 feature set from the Waber-Cromer scale and PC-scores of both scales using decision trees classify CdSe as Z. One of the reasons that AB compounds with A = Cd show difficulty in our classification could be attributed to the *omission* of *d*-orbital radii in our feature set. Atom- and orbital-projected density of states (PDOS) spectra for CdSe in W and Z-structures show that the top of the valence band is comprised of 4*d*-states, indicating its dominant role in chemical bonding (see Supplementary Note 3 and Supplementary Figure 2). These 4*d*-states reduce the band gap, thereby increasing the covalent character or bond polarizability, which is not readily captured by the ML model from the given training set. To capture the effect of Cd 4d-states on the band gap, we considered MgTe and CdTe. We calculated the band gap of MgTe in W-structure to be 2.4 eV. For the exact same crystal structure (without relaxing the internal coordinates and unit cell geometry), we substituted Cd-atom in the place of Mg-atom and re-calculated the band gap to be 0.8 eV.

The compound MgTe was also misclassified in our classification learning. Note that Saud *et al.* also reported difficulty in classifying it, even though they used an exhaustive list of feature sets (9 features) and explored a completely different set of ML methods. Our DFT calculations reveal that the energy difference between W–Z and W–R structures in MgTe are of the order of 3.8 and 2.6 meV/f.u., respectively, indicating that MgTe lies on the borderline, making it difficult for our ML methods to accurately assess its relative position in the phase space.

**Figure 6:**
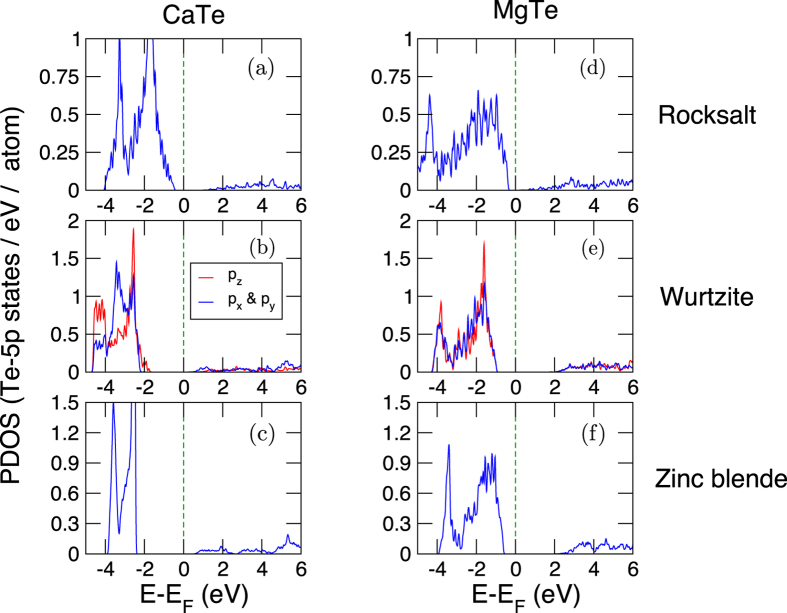
Te-atom 5*p*-orbital projected density of states (PDOS). (**a**–**c**) CaTe and (**d**–**f**) MgTe in Rocksalt, Wurtzite, and Zinc blende structures. In CaTe the ground state is Rocksalt, whereas in MgTe it is Wurtzite. In Rocksalt and Zinc blende structures, for both compounds, the three 5*p*-orbitals are degenerate. However, the 5*p*-orbital splitting (into 5*p*_*z*_ and 5*p*_*x*,*y*_) is more pronounced in CaTe, relative to MgTe. Data shown for DFT-PBEsol calculations using ultrasoft pseudopotentials.

Why is MgTe more difficult to classify using ML methods? In our data set, there are a total of six AB compounds with Te-atom in the B-site. Among the six, three of them (BeTe, CdTe, and ZnTe) have Z, two of them (CaTe and SrTe) have R and MgTe has W ground state structure. We now examine the electronic structure of MgTe with CaTe for the three crystal structures (Fig. 6). CaTe is an ideal choice, because of the similarity in the valence electron configuration between the Group 2 elements: Mg with ([Ne]3*s*^2^) and Ca with ([Ar]4*s*^2^). The ground state for CaTe was determined to be the R-structure, in agreement with Saud *et al.*; the energy difference between R–W and R–Z is 302 and 621 meV/f.u., respectively.

Noticeable differences in the partial-densities of states (PDOS) are found in the bandwidth of Te 5*p*-orbitals for the two compounds, where they are relatively narrow in CaTe compared to the broader spectral features in MgTe. In the R- (Fig. 6a,d) and Z-structures (Fig. 6c,f), where all Ca-Te and Mg-Te bond lengths are equidistant, the electronic states of 5*p*_*x*_, 5*p*_*y*_, and 5*p*_*z*_ orbitals overlap. Key differences occur in the W-structure (Fig. 6b,e), where the 5*p*-orbitals splits into 5*p*_*z*_ and 5*p*_*x*,*y*_ (we use the notation 5*p*_*x*,*y*_ to denote the fact that 5*p*_*x*_ and 5*p*_*y*_ overlap). Furthermore, the difference in the centers of masses between 5*p*_*z*_ and 5*p*_*x*,*y*_ are also more pronounced in the two compounds for the W-structure. These subtle changes in the electronic structure affect the energetics, thereby favoring one structure over the other. We conclude that the orbital radii scales (from both Chelikowsky and Waber-Cromer) are probably insensitive to the relative energetic competition (and orbital splitting) of Te 5*p*_*x*_, 5*p*_*y*_, and 5*p*_*z*_-states seen in MgTe relative to the other five ATe compounds, which eventually favors the R- or Z-structure. We conjecture that this problem could be potentially alleviated by adding more AB compounds that behave similar to MgTe, but remains to be explored further.

To summarize this section on AB compounds, we have shown that PCA can be used to construct linear combinations of orbital radii. The classification performance of these linear combinations from PCA with respect to [*r*_*σ*_, *r*_*π*_] is an important outcome of our work with potential implications for ML beyond binary wide band gap AB compounds. It is also important to recognize that orbital radii can themselves serve as the data matrix for ML (without the need for considering the linear combinations). One of the problems with such a feature set is that these orbital radii could show a high degree of statistical correlation (as seen in Fig. 3), indicating redundancy of information. PCA removes the correlation (redundancy) by finding linear combinations of tightly connected orbital radii and the PCs (eigenvectors) are orthogonal to one another (see Fig. 5). The orthogonality condition ensures that the features are independent (under the assumption that the physical process is governed by Gaussian or Normal distribution), which in turn allows us to explore a broad range of ML methods without compromising the interpretability or performance.

### Mechanical Properties Classification of Intermetallic RM compounds

We now classify the mechanical properties of intermetallic RM compounds, where R and M represent chemistries of rare-earths and main group or transition metal elements, respectively. Unlike the AB compounds (discussed earlier) that have wide band gap and no partially filled *d*- or *f*-like orbitals in their valence electron configuration, these RM compounds are metallic with delocalized electrons; the *d*- and *f*-like orbitals control the electronic and magnetic states. Furthermore, these **B2**-RM intermetallics have unique mechanical properties; normally, intermetallic compounds are brittle (*i.e.*, no plastic deformation observed in the stress-strain curve); however, Gschneidner *et al.*^31^ discovered a family of RM intermetallics that were experimentally found to show high ductility and high fracture-toughness at room temperature. One of the intriguing aspects of these materials is that not all RM intermetallics in **B2** structure-type are ductile; some of them are also brittle, indicating a delicate balance in the structure-chemistry-property relationships.

Previous plane-wave pseudopotential-based DFT calculations^29^ have shown the relative importance of the electronic states of M-atoms near the Fermi level (*E*_*F*_) to classify the ductile and brittle mechanical behavior. From atom- and orbital-PDOS, it was found that in ductile RM compounds there are no bands of *p*- or *d*-orbitals of the M-atom that exhibit directional (anisotropic) character near the *E*_*F*_. It was concluded that a given RM is expected to be ductile when the valence electronic states of M-atoms are primarily *s*-like with *d*-bands located at 1 eV or more below the *E*_*F*_. The objective of this work is to use orbital radii scales, construct their linear combinations, and apply ML methods to uncover classification rules that separate ductile and brittle RM intermetallics. We ask the following questions: *Could orbital radii and ML methods capture the physics described by DFT calculations with substantially less computational overhead and complexity?* Can this undertanding be used to predict new and previously unexplored ductile RM intermetallics for experimentation?

**Figure 7:**
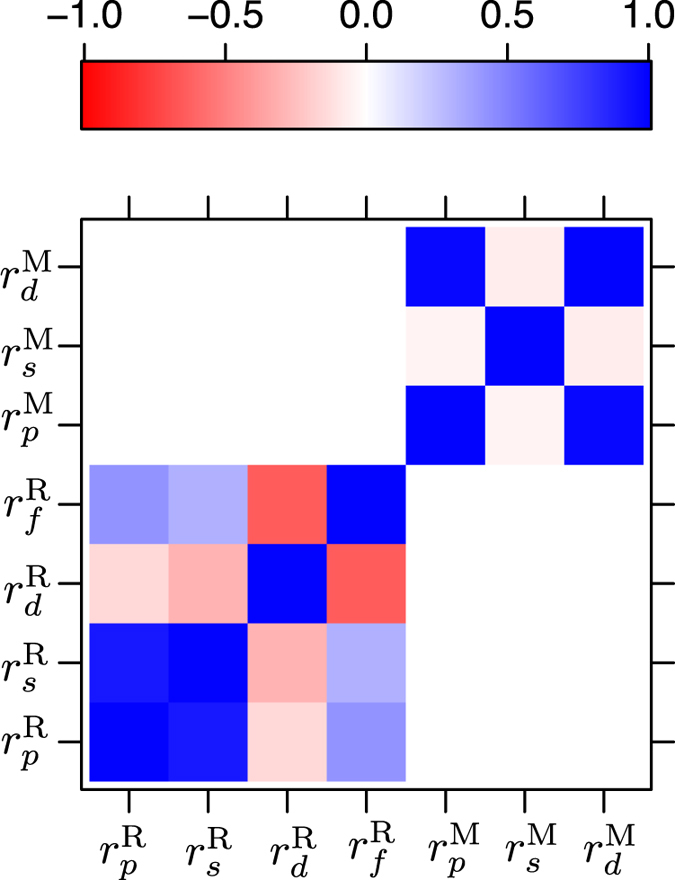
Correlation plot of 143 RM intermetallics based on the Waber-Cromer orbital radii scale. Superscripts R and M indicate the orbital radii data for rare-earth and main group or transition metal elements, respectively. Subscripts *s, p, d*, and *f* indicate the orbital character of the radii (*r*). Red (negative) and blue (positive) colors indicate strong inverse and direct correlation, respectively, whereas white color indicates no correlation.

In Fig. 7, the correlation plot of the Waber-Cromer orbital radii for the 143 RM compounds is shown. As noted before we do not use the Chelikowsky scale for this problem, because the radii are not given for rare-earth elements. Notice the strong statistical correlation seen in the orbital radii of R- and M-elements, similar to Fig. 3 for AB compounds. For the R-atom, 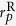 and 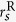 show direct or positive correlation, whereas the 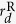 and 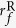 show inverse or negative correlation. In the case of the M-atom, the 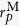 and 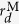 show strong positive correlation and are not linearly related to 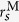. Since strong statistical correlation implies redundancy of information, we also apply PCA to construct their linear combinations. As a result, we built two separate data sets for classification learning: one based on the raw orbital radii data alone, and in the other, we performed PCA to construct linear combinations of orbital radii. Unlike the AB compounds, for which we knew *a priori* about *r*_*σ*_ and *r*_*π*_, there are no such linear combinations for RM compounds in the literature. We construct these linear combinations for the first time from PCA.

In Table 5, the linear combination of orbital radii (PC’s) and % variance explained for each of those combinations are given. The first four PC’s together capture ~94% variance in the data set and we focus our attention there. Notice that RM-PC1 and PC3 contain linear combinations of orbital radii of only R-elements. In RM-PC1, the effect of 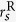 and 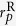 orbital radii is pronounced, whereas in RM-PC3 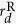 dominates; in both RM-PC1 and PC3, the effect of 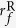 is non-trivial; it is unlikely that the functional form could have been surmised. On the other hand, RM-PC2 and PC4 contain linear combinations of orbital radii of only M-elements. More specifically, RM-PC2 captures the contributions from 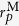 and 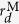 and RM-PC4 captures the role of 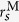 orbital radii. From Table 5, we infer that in the given data set there is no mixing of orbital radii of R and M elements. We also note that the family of **B2** intermetallics extends beyond the RM chemistries considered in this work (*e.g.*, NiTi). When one constructs a comprehensive data set with all known material **B2** structure-types (including the RM compounds), then one may be able to discover some orbital mixing.

**Figure 8:**
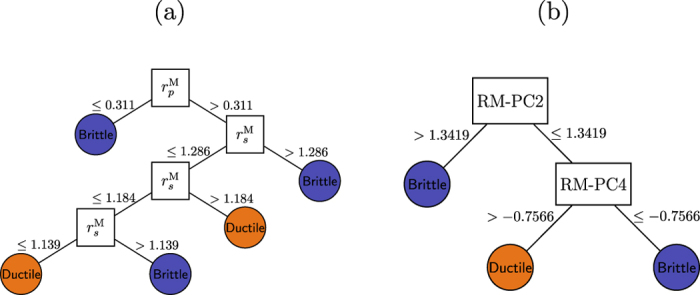
Decision trees for classifying ductile and brittle B2 RM intermetallics based on the full training set. (**a**) Using raw Waber-Cromer orbital radii data and (**b**) after PCA to obtain their linear combinations. In (**a**) we found that 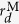 could substitute for 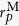 at the root node in Fig. 8a without any loss in the classification accuracy, because they are linearly correlated (see Fig. 7). (**b**) The meaning of RM-PC2 and RM-PC4 is given in Table 5.

After correlation analysis and PCA, we now focus our attention on classifying the ductile and brittle mechanical properties of the 30 known RM intermetallics. We utilize decision trees and SVM for classification learning. With decision trees, we obtain classification accuracies of 86.7% and 96.7% based on LOO-CV and full training set, respectively, with the raw orbital radii scale (without any linear combinations). On the other hand, we attain marginally better classification accuracy of 90% from LOO-CV with the linear combinations of orbital radii from PCA. The performance of SVMs (for various combinations of feature sets) were comparable to that of the decision trees, where we attained classification accuracies of 86.7 and 93.3% based on LOO-CV and full training set, respectively, with the raw orbital radii scale. Similarly, with the linear combinations from PCA we obtained accuracies of 86.7% and 90% based on LOO-CV and full training set, respectively. In Fig. 8a,b, the decision trees from raw orbital radii scale and PCA, respectively, are shown.

Interestingly, both decision trees identify features associated with *only* M-elements to be critical for ductile and brittle mechanical property classification—in excellent agreement with previous DFT calculations^29^. The conditions 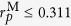 and RM-PCA > 1.3419 in Fig. 8a,b, respectively, for the brittle property correspond to RM intermetallics that have Zn- and Mg-atoms in the M-site. This result is consistent with Gschneidner *et al.*, who also suggested that Zn- and Mg-based compounds should exhibit similar mechanical behavior. Moreover, it was suggested that Cd-compounds are also expected to behave similar to Zn- and Mg-based compounds. However, we differ in our interpretation for the YCd compound; our analysis indicates some form of interaction between 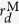 (or 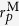) and 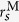 orbitals, and thus *different* deformation properties.

A classification accuracy of <100% indicates that there are misclassified instances (see Table 6). The ScIr compound was misclassified as ductile by both decision trees and also SVM; additionally, ScCo and YCd were also misclassified as ductile by the decision tree shown in Fig. 8b that uses the linear combinations obtained from PCA. SVM also identifies ScCo and YCd as misclassified instances.

**Figure 9:**
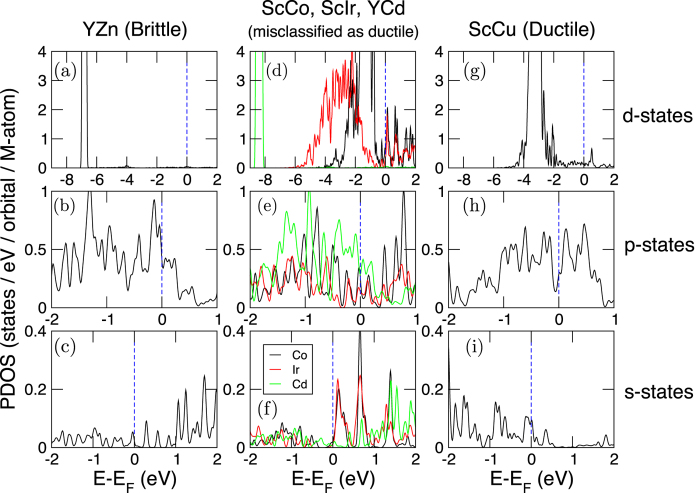
Atom- and orbital-PDOS of the *M*-atom for *d*-, *p*-, and *s*-orbitals in RM compounds. (**a**–**c**) Zn *d*-, *p*-, and *s*-states, respectively, in brittle YZn (**d**–**f**) Co (black), Ir (red) and Cd (light green) *d*-, *p*-, and *s*-states, respectively, in ScCo, ScIr, and YCd. Our decision tree algorithm misclassifies these three compounds as ductile, although experimentally Gschenidner *et al.* report them as brittle. (**g**–**i**) Cu *d*-, *p*-, and *s*-states, respectively, in ductile ScCu. Blue dotted line indicates the position of the Fermi level (*E*_*F*_). Data shown for DFT-PBE calculations using projector augmented-wave pseudopotentials.

We performed DFT calculations for the three misclassified compounds (ScIr, ScCo, and YCd) and compared their electronic structures to compounds that were correctly identified by our classification learning model to be ductile (ScCu) and brittle (YZn). In Fig. 9, we show the orbital-PDOS for the *d*-, *p*-, an*d s*-states of the M-atom for brittle (Fig. 9a–c), misclassified (Fig. 9d–f), and ductile (Fig. 9g–i) compounds. In brittle YZn, clearly, the Zn-*p*-states (Fig. 9b) dominate the *E*_*F*_; the *d*-states (Fig. 9a) are fully occupied and are at ~7 eV below the *E*_*F*_. On the other hand, in ductile ScCu the Cu-*d*-states (Fig. 9g) are located between 2–4 eV below the *E*_*F*_ and there are some contributions from *p*- (Fig. 9h) and *s*-states (Fig. 9i) at the *E*_*F*_. These results agree well with those of Gschneidner *et al.*, although that reference does not report the local *s*- and *p*-states.

In the case of misclassified ScCo, there are more Co-*d* states near the *E*_*F*_ (Fig. 9d) relative to the Cu-analogue. The center of mass of the Co 3*d*-band is also shifted more towards the *E*_*F*_; the spectral signatures and peak positions of *p*- and the *s*-orbital states (in Fig. 9e,f, respectively) are comparable to that of the Cu-analogue. These electronic structure results, in conjunction with the rules given by Gschneidner *et al.*, appear to indicate that ScCo is probably brittle. If this result holds true, then our classification model is genuinely misclassifying the ScCo compound.

With ScIr (Fig. 9d–f), the bandwidth associated with the Ir 5*d*-orbitals is found to be larger (due to its spatially extended nature) and its center of mass is shifted away from the *E*_*F*_ (relative to the Co-analogue). At the same time, in the 0 to 1 eV range (above *E*_*F*_), the 5*d*-spectral features resemble those of the Co-analogue. Similarly, the Ir *p*- and *s*-states in ScIr resemble those of the ScCo electronic structure. Note that we do not account for electron-correlation or spin-orbit coupling in our calculations. From our own DFT calculations and applying the rules of Gschneidner *et al.*, it is not obvious whether ScIr is ductile or brittle.

Unlike a phase transition (e.g. ferroelectricity, where the order parameter is polarization), which is accompanied by a change in symmetry of the order parameter, here we are not dealing with any similar phase change concomitant with ductile and/or brittle behavior. In our case, either an RM intermetallic is ductile or brittle, and therefore, we cannot write a phenomenological free energy expansion in terms of the order parameter. Alternatively, we can employ the recently developed mesoscale dislocation mechanics^32^ that uses energy-based stability criterion to validate our misclassifications. According to this approach, the necessary and sufficient crystallographic conditions that must be satisfied by a **B2** material for enhanced ductility are that 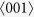 should be the dominant slip direction, yet 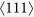 slip should also be possible with the formation of 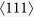 anti-phase boundaries (APBs) and APBs should have bistable existence on both 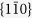 and 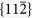 planes, respectively. The notation 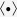 and 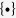 follow the conventions of Miller indices for representing families of crystallographic equivalent directions and planes, respectively. Detailed investigation on a range of **B2** materials revealed that elastically anisotropic **B2** alloys do not satisfy the necessary and sufficient conditions; hence they are brittle. On the other hand, ductile **B2** alloys are nearly isotropic. Sun and Johnson suggested the use of the Zener anisotropy ratio \ [A 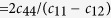, where *c*_11_, *c*_12_, and *c*_44_ are the elastic constants of the **B2** cubic structure], as a *qualitative* figure of merit to classify the mechanical properties^32^. In *ductile* **B2** systems (similar to those explored in this paper), the value of A should be close to one^32^. Wang *et al.*^33^ have calculated the A-ratio for ScIr as 1.584 from DFT calculations (using GGA approximation), which is closer to that of the ductile ScCu (1.5) as opposed to that of the brittle YZn (1.985). Based on the A-ratio, we validate our findings for ScIr and predict it to be ductile. Therefore, we recommend experimental re-evaluation of its mechanical properties.

In the case of YCd, its electronic structure is found to be similar to that of YZn. Furthermore, the A-ratio for YCd is reported as 2.894, which is much larger than that of YZn (1.985) indicating that it is probably brittle. Unfortunately, Wang *et al.* do not report the A-ratio of ScCo compound for us to compare. We also note that Gschneidner *et al.* have not reported any quantitative mechanical experiments data on the three misclassified materials.

The reasons for observing misclassifications in our ML could be attributed to the following factors: (i) small size of the data set, (ii) insufficient examples of materials in our training set that resemble the mechanical properties of ScCo, ScIr, and YCd compounds, and/or (iii) the Waber-Cromer orbital radii may not necessarily contain the physics representative of the complex electronic structures of ScCo, ScIr, and YCd. Strictly, we recommend re-evaluation of the mechanical properties of ScCo, ScIr and YCd before considering the application of our results for designing new materials. Nonetheless, based on the fact that our ML approach identifies key electronic structure features that agree strongly with the state-of-the-art DFT calculations, gives us some confidence in applying the classification rules shown in Fig. 8 to predict the mechanical properties of the remaining unexplored 113 RM compounds. Note that our classification rules are applicable only for RM compounds with **B2** crystal structure-type and we have assumed all 113 RM compounds to have **B2** crystal structure-type, which may not be necessarily true. We predict 57 out of 113 compounds to be ductile from Fig. 8a that have M = Cu, Ni, Au, Ag, Pd, Pt and Ir. Similarly from Fig. 8b, 77 compounds are predicted to be ductile. As discussed earlier, in addition to M = Cu, Ni, Au, Ag, Pd, Pt and Ir, even M = Cd and Co compounds are predicted to be ductile from Fig. 8b.

## Discussion

We have shown how to construct linear combinations of orbital radii solely from data without any *a priori* assumption about their functional form. We demonstrated our ML approach on two broad materials classes that include insulators (AB compounds) and metallic materials (RM intermetallics). We first tested the performance of PCA-derived linear combinations in classifying the crystal structures of AB compounds and found that they perform equally well as the canonical *r*_*σ*_ and *r*_*π*_ feature sets. We identified misclassified instances and provided a rationale for such occurrences (in the process we addressed some of the potential shortcomings of orbital radii scales and small data sets, in general).

We then extended the ML principles gleaned from relatively simple AB compounds, to more electronically complex RM intermetallics with the objective of classifying mechanical deformation behavior. We found excellent agreement between the classification rules extracted from ML and insights from DFT calculations. We identified that the behavior of Cd-based RM compounds could differ from that of the Zn- and Mg-based compounds. Previous DFT work predicted similar behavior among Cd-, Zn- and Mg-based compounds^29^. Although the accuracies of our ML models were not 100%, we identified ScIr (originally considered as a brittle material) to be potentially ductile, requiring re-evaluation of its mechanical properties. We also predicted several new ductile RM intermetallics using classification learning. It was interesting to find that the orbital radii features of atom-R were not identified to be critical for classifying ductile from brittle RM compounds, particularly when our DFT+*U* calculations for the ductile DyCu compound showed evidence for high density of Dy 4*f*-states at the Fermi level (see Supplementary Note 3). This finding could have important implications for targeted materials design, because we now have an additional degree of freedom to dope other rare-earth elements at the R-site without affecting the mechanical properties of the parent alloy. Broadly, our informatics work shows how by leveraging available small data sets, ML methods, and accurate electronic structure calculations it is possible to enable materials discovery; the concepts described here can be readily applied beyond the binary systems and properties explored in this paper.

We clarify the relevance of our informatics approach at a time when high-throughput first principles calculations, such as those found in The Materials Project^34^ and AFLOWLIB^35^, have attracted significant attention. We are fully aware that one could employ accurate *ab initio* calculations to evaluate the relative energetics of simple compounds, such as the wide band gap AB alloys explored in this work. In fact, we have shown in this paper that classification learning, indeed, achieves accuracy comparable to first principles based methods. Our work reinforces data-driven informatics based learning as an alternative paradigm to high-throughput first principles calculations for rapidly identifying new and previously unexplored material compositions with targeted properties. This crucial finding makes our approach highly attractive for the computational design of complex materials with defects, solid solutions and multicomponent alloys, whose structures and compositions are not reported in online repositories such as International Crystal Structure Database (ICSD)^36^. Furthermore, we also utilize experimental data (where available) as the starting point for our classification learning, as demonstrated using RM intermetallics, which is a departure from the high-throughput literature where databases from first principles calculations are frequently mined. Clearly, in our approach the accuracies of the reported experimental data in the literature are critical towards assessing the success of our ML models (similar to the importance of the accuracies of density functional theory or other theories in computing properties within the high-throughput framework). We also acknowledge that our reliance on experimental data introduces additional complexities for ML in the form of handling small or tiny data sets and uncertainties in error measures, which we have shown can be addressed by employing physically meaningful feature selection/extraction methods, cross-validation schemes and *ab initio* calculations of misclassified instances. We believe that to achieve accelerated materials design and discovery, it is crucial to consider complexities and uncertainties associated with data coming from experiments and we note that the ML approaches described in this paper are a significant step in that direction.

## Methods

### Machine Learning (ML)

Classification is a machine learning (ML) approach that separates data into pre-defined classes. In classification, we have a data matrix **X** of dimension *m* × *n*. The rows of **X** are *m* chemical compositions and columns are *n* features. Each chemical composition in **X** belongs to a class specified by another categorial attribute (*Y*) called the class label, *Y* = (*y*_1_, *y*_2_, …, *y*_*m*_) with *p* distinct labels 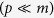. The source for class labels could be either experiments or high-fidelity computations. In this particular case, the classification problem is referred to as *supervised learning*, because the class label attribute (*Y*) is a part of the data set. A subtlety to this description is that the column vector *Y* could also represent a numeric attribute, in which case the supervised learning is referred to as *regression* or *prediction*. The objective of supervised-ML is to find a function, 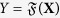 that maps features (matrix **X**) onto *Y*. The mapping functions determine the decision boundaries that help separate one class (*y*_*i*_) from the other(s). Our interest in supervised learning is motivated from earlier studies^37^,^38^,^39^,^40^,^41^, which showed that ML can be used for predicting new materials with specific functionalities in an accelerated manner. In this work, we use orbital radii as features to which ML is applied.

The three ML methods we use are principal component analysis (PCA)^30^, decision trees^42^, and support vector machines (SVM)^43^. PCA is used for constructing the linear combinations of orbital radii; decision trees and SVM are used for supervised classification learning. These classification ML methods determine the functional relationship, 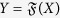. While decision trees partition the data in a linear fashion using vertical and horizontal lines, SVM is a non-linear method. We use the R-package for PCA^44^. Decision trees are performed using the J48 algorithm^45^ as implemented in the Weka platform^46^ and with the default values of the hyper-parameters. SVMs are performed using the RBF-kernel as implemented in the scikit-learn python module^47^, and the hyper-parameters are optimized through cross-validation. Regarding the choice of kernels for SVM’s, the RBF-kernel is standard practice relative to other popular kernels (such as linear and polynomial), and has the advantage that it is “universally consistent”, which means that with enough data and appropriate choice of kernel hyper-parameters, the SVM RBF-kernel can find the Bayes optimal classifier for any distribution^48^. This is not to say that it will be the optimal classifier all the time, the “no-free-lunch” theorem prevents one from making such a statement^49^.

Classification accuracies are calculated as the ratio of number of compounds correctly classified to the total number of compounds that are used for training. We trained the decision tree and SVM algorithms in two ways: (i) on the full training set and (ii) with leave-one-out cross validation (LOO-CV). The LOO-CV approach involves training the ML algorithm on all but one data point and then applying that classifier to the left-out data point. The process is repeated until each compound is left out exactly once and its crystal structure or properties *predicted* from the knowledge of the remaining compounds. LOO-CV is a common procedure used in ML when the training data sets are smaller in size. Additional details necessary to reproduce the ML are given in Supplementary Note 4.

### Density Functional Theory

Density functional theory (DFT) calculations were performed within the generalized gradient approximation (GGA) as implemented in Quantum ESPRESSO^50^. For the AB solids, the PBEsol exchange-correlation functional^51^ was used and the core and valence electrons were treated with ultrasoft pseudopotentials^52^. The Brillouin zone integration was performed using a 12 × 12 × 12 Monkhorst-Pack *k*-point mesh^53^ centered at Γ and 60 Ry plane-wave cutoff. For modeling the RM intermetallics, the PBE exchange-correlation functional^54^ was used. The core and valence electrons were treated with projector augmented-wave (PAW) pseudopotentials^55^. We chose these exchange-correlation functionals and pseudopotentials to directly compare our results with available electronic structure calculations^29^. All RM compounds were treated as spin unpolarized except those containing Dy. For the Dy-atom, we used the PAW pseudopotential generated by Topsakal and Wentzcovitch^56^ that explicitly treats the 4*f*-orbitals as valence states. Collinear ferromagnetic spin order was imposed on the Dy-atom. We compared the electronic structure of the intermetallic compound with and without Hubbard-*U* correction on the Dy *manifold*; we chose a *U*_*f*_ value of 5 eV^56^. (see Supplementary Note 3 and Supplementary Figure 3). For the DFT+*U* calculations, the standard Dudarev implementation^57^ was used. In compounds that do not contain Dy, non spin-polarized calculations were performed. The Brillouin zone integration was performed using a 10 × 10 × 10 Monkhorst-Pack *k*-point mesh^53^ centered at Γ and 60 Ry plane-wave cutoff.

For both AB and RM compounds, to yield optimally smooth pseudopotentials, we used the Troullier-Martins pseudization method^58^. The scalar relativistic pseudopotentials were generated using the atomic package^50^ with the inclusion of nonlinear core corrections. For the density of states calculations, 14 × 14 × 14 Monkhorst-Pack *k*-point mesh centered at Γ was used. The atomic positions and the cell volume were allowed to change until an energy convergence threshold of 10^−8^ eV and Hellmann-Feynman forces less than 2 meV/Å, respectively, were achieved. The space groups of the optimized structures were determined using FINDSYM^59^ and the resulting crystal structures were visualized in VESTA^60^.

## Additional Information

**How to cite this article**: Balachandran, P. V. *et al.* Materials Prediction via Classification Learning. *Sci. Rep.* **5**, 13285; doi: 10.1038/srep13285 (2015).

## Supplementary Material

Supplementary Information

## Figures and Tables

**Table 1:** Percentage variance explained (in descending order) by the eigenvalues of Chelikowsky and Waber-Cromer orbital radii scales.

PCs	Chelikowsky (C) Scale (%)	Waber-Cromer (WC) Scale (%)
PC1	53.48	51.79
PC2	44.38	27.17
PC3	2.08	20.98
PC4	0.06	0.06

PC stand for principal components (eigenvectors).

**Table 2:** Linear combinations of orbital radii from PCA for Chelikowsky (C) and Waber-Cromer (WC) scales.

Chelikowsky (C) Scale	Waber-Cromer (WC) Scale
C-PC1 = 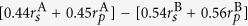	WC-PC1 = 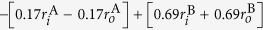
C-PC2 = 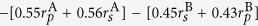	WC-PC2 = 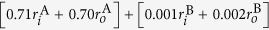
C-PC3 = 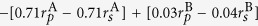	WC-PC3 = 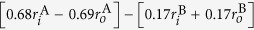
C-PC4 = 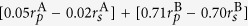	WC-PC4 = 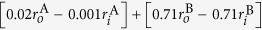

PC refers to principal component. The 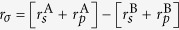 and 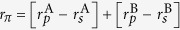.

**Table 3:** Accuracy (in terms of % correctly classified) of AB compounds using both orbital radii scales.

Method	Chelikowsky		Waber-Cromer	
	*r*_*σ*_, *r*_*π*_	PCA	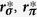	PCA
Trees	96.4%, 83.6%	96.4%, 85.5%	94.5%, 85.5%	92.7%, 83.6%
SVM	96.4%, 96.4%	94.5%, 90.9%	100%, 96.4%	100%, 94.5%

The first and second values for each column in the table report the % accuracy based on full training set and leave-one-out cross-validation (LOO-CV) method, respectively.

**Table 4:** Summary of misclassified AB compounds by using the full training set and their lowest energy structures from our DFT-PBEsol calculations using ultrasoft pseudopotentials.

AB	Saud *et al*. (original label)	Chelikowsky		Waber-Cromer		DFT
		*r*_*σ*_, *r*_*π*_	PCA	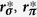	PCA	
CdO	R	R, R	R, **W**	**W**, **W**	**W**, R	R
CdS	W	**R**, W	W, **Z**	W, W	**Z**, W	W
CdSe	W	W, W	**Z**, **Z**	**Z**, W	**Z**, W	**Z**
CdTe	Z	**W**, Z	Z, Z	Z, Z	Z, Z	Z
MgTe	W	W, W	**Z**, **Z**	**R**, **Z**	**Z**, **Z**	W

R, W, and Z stand for rocksalt, wurtzite and zinc blende structures, respectively. Structure labels as reported in Saud *et al.*^25^ are also given. Structures labeled using bold font indicate misclassifications with respect to the Saud *et al.* label. The two entries separated by a comma under each scale and feature set represent prediction from decision trees (first) and SVM (second). Note that our DFT calculations identify CdSe as Z, which was originally labeled as W in Saud *et al.*

**Table 5:** Linear combinations of orbital radii from PCA for the RM intermetallics using Waber-Cromer scale and % variance explained by each of those linear combinations.

Linear combinations of orbital radii	% variance explained
RM-PC1 = 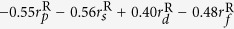	34.22
RM-PC2 = 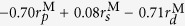	28.35
RM-PC3 = 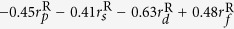	16.86
RM-PC4 = 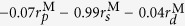	14.20
RM-PC5 = 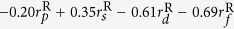	5.64
RM-PC6 = 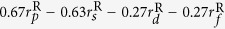	0.43
RM-PC7 = 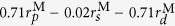	0.30
	**Σ =** **100**

Σ is the total variance explained by 7 principal components (PC’s). In general, each PC is denoted as RM-PC*i* = 
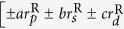

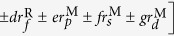
 , where *i* varies from 1 to 7 and *a*–*g* are the weighted coefficients of each orbital radii for a given PC. In the table, for improved readability, we do not show those orbital radii whose coefficients are zero. We can infer that RM-PC1, PC3, PC5 and PC6 capture only the features of atom-R 
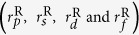
 whose coefficients for the features of atom-M 
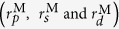
 are zero, whereas RM-PC2, PC4 and PC7 capture only the features of atom-M.

**Table 6:** Summary of most commonly misclassified RM compounds from classification learning.

RM	Gschneidner *et al.*(original label)	Waber-Cromer	
		*r*^R^, *r*^M^	PCA
ScCo	Brittle	Brittle	**Ductile**
ScIr	Brittle	**Ductile**	**Ductile**
YCd	Brittle	Brittle	**Ductile**
