# Supplementary material for: Materials Prediction via Classification Learning
Source: Sci Rep. 2015 Aug 25;5:13285. doi: 10.1038/srep13285 (PMC4548442; doi:10.1038/srep13285)
Supplement: Supplementary Information [file srep13285-s1.pdf]

SUPPLEMENTAL MATERIAL FOR

Materials Prediction via Classification Learning

Prasanna V. Balachandran,[1] James Theiler,[2] James M. Rondinelli,[3] and Turab Lookman[1,*]

[1] *Theoretical Division, Los Alamos National Laboratory, Los Alamos, NM 87545, USA*
[2] *Intelligence and Space Research, Los Alamos National Laboratory, Los Alamos, NM 87545, USA*
[3] *Department of Materials Science & Engineering, Northwestern University, Evanston, IL 60208, USA*

(Dated: June 26, 2015)

SUPPLEMENTARY NOTE 1

ENERGETICS OF SIX AB COMPOUNDS

Note that in Saad *et al.*, six (out of 55) AB compounds were originally labeled as dual structures, where the ground state structures were designated as borderline due to close energetic competition. We performed DFT calculations on six compounds that were originally labeled as dual structures and re-labeled them uniquely to a single structure-type based on the phase with the lowest energy (amongst the three R (Rocksalt), W (Wurtzite), and Z (Zinc blende) structures). DFT energetics data are given in Supplementary Table 1.

Supplementary Table 1 | Total energy (in eV) for AB compounds normalized with respect to the lowest energy configuration. Column with 0 eV is predicted as the lowest energy structure-type for the AB compounds from DFT-PBEsol calculations using ultrasoft pseudopotentials.

Compound	Z	W	R
SiC	0.0	0.009	2.64
CdS	0.006	0.0	0.341
ZnS	0.0	0.012	1.095
ZnSe	0.0	0.018	1.037
MgS	0.326	0.288	0.0
MgSe	0.204	0.181	0.0

SUPPLEMENTARY NOTE 2

ORBITAL RADII SUM VS. SHANNON IONIC RADII

Scatter plots showing linear relationships between between Chelikowsky orbital radii sum $[r_s{+}r_p]$ for the A-(Supplementary Figure 1a) and B-atoms (Supplementary Figure 1b) and their corresponding Shannon ionic radius in the 6-fold coordination.

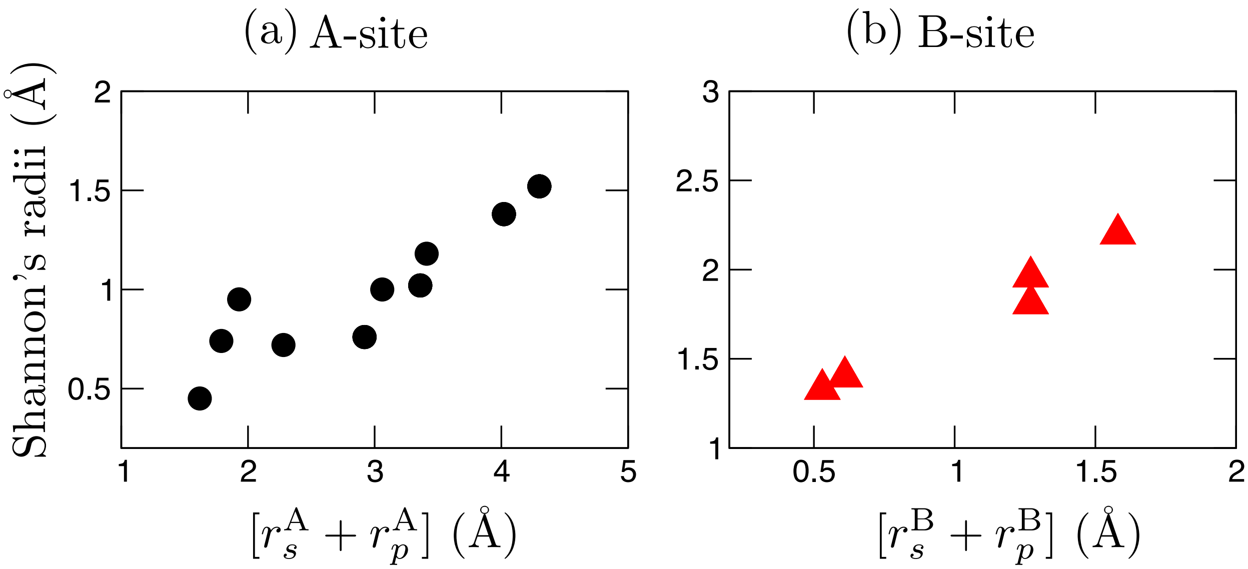

Supplementary Figure 1 | (Color online) **Linear relation between Chelikowsky orbital radii sum and Shannon's ionic radii in** AB **compounds** (a) A-site that include divalent (Be, Mg, Ca, Sr, Zn, and Cd) and monovalent cations (Li, Na, K, and Rb) and (b) B-site that include anions (O, F, Cl, Br, and I). For the Shannon ionic radii, we used 6-fold coordination.

SUPPLEMENTARY NOTE 3

PROJECTED DENSITY OF STATES

CdSe

Atom- and orbital-projected density of states (PDOS) spectra for CdSe in W Supplementary Figure 2a and Z-structures Supplementary Figure 2b show that the top of the valence band is comprised of $4d$-states, indicating its dominant role in chemical bonding. These $4d$-states impacts the band gap, thereby increasing the covalent character or bond polarizability.

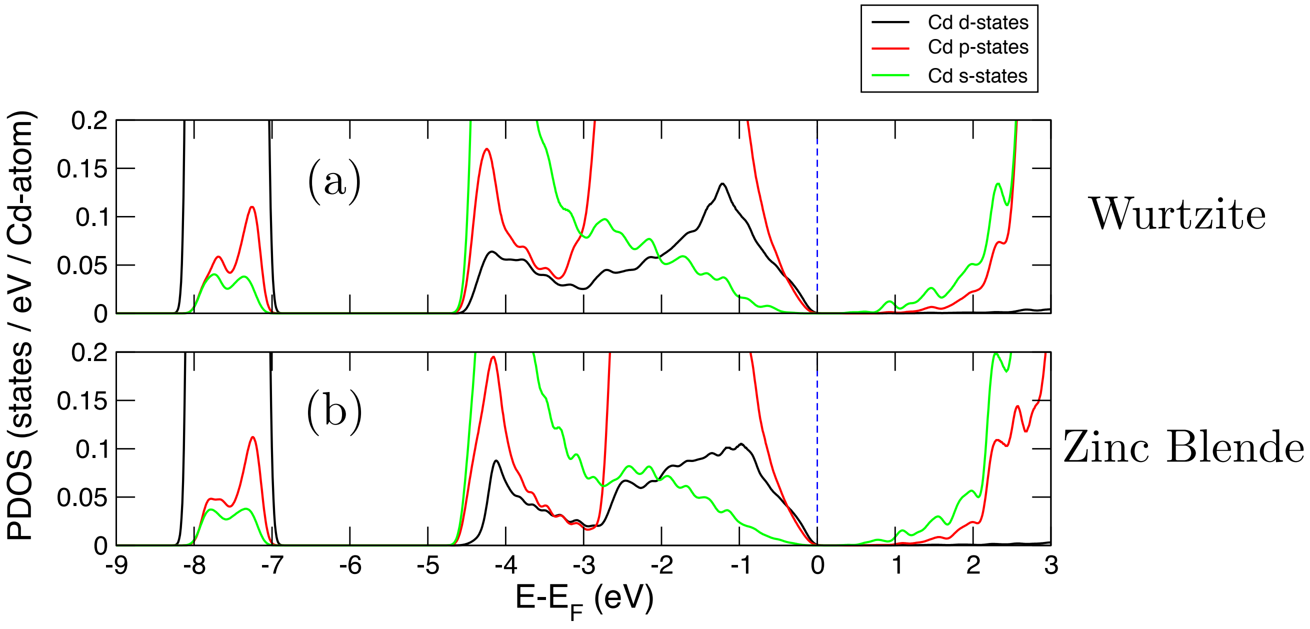

Supplementary Figure 2 | (Color online) **Cd-atom orbital projected density of states (PDOS) in CdSe.** (a) Wurtzite and (b) Zinc blende structures. Notice the significant contributions from the spectral features of $4d$-states near the Fermi level, E_F (dash vertical blue line). Data shown for DFT-PBEsol calculations using ultrasoft pseudopotentials.

Dy-$4f$ states in the electronic structure

It is interesting to note that our analysis reveals the orbitals of rare earth elements do not affect the mechanical behavior. The previous DFT work of Gschneidner *et al*[1] did not explicitly treat the $4f$-states of the rare earth elements. To understand this observation, we calculate the electronic structure of DyCu system and probe the effect of the partially filled $4f$-electronic states of the Dy-atom. We use the DFT+U method to treat the strong electron-correlation of $4f$-orbitals and included an effective Hubbard-U_f value of 5 eV.[2] Although, we note that dynamical mean field theory (DMFT) may be more suited for the DyCu system.[3] We also imposed collinear ferromagnetic spin order on the Dy-atom and found that the DyCu system (with U_f = 5 eV) in the spin-polarized ferromagnetic state was 5 eV/f.u lower in energy than that of the non spin-polarized calculation. In Supplementary Figure 3a and b, the Dy-atom $4f$-orbital PDOS without and with the $+U$ correction is shown, respectively. At the DFT level (Supplementary Figure 3a), we find three distinct $4f$-peaks in the immediate vicinity of the E_F. With U_f = 5 eV (Supplementary Figure 3b), the $4f$-states in the spin-up channel shift ∼2 eV lower in energy. More pronounced changes in the spectral features are seen in the spin-down channel.

The three $4f$-peaks in the spin-down channel split from one another, with one of them remaining pinned at the E_F, another moving ∼3.5 eV below the E_F, and the third peak moving ∼3 eV above the E_F. Our DFT calculations suggest an important role of $4f$-states near the E_F, which was originally not considered in the calculations reported by

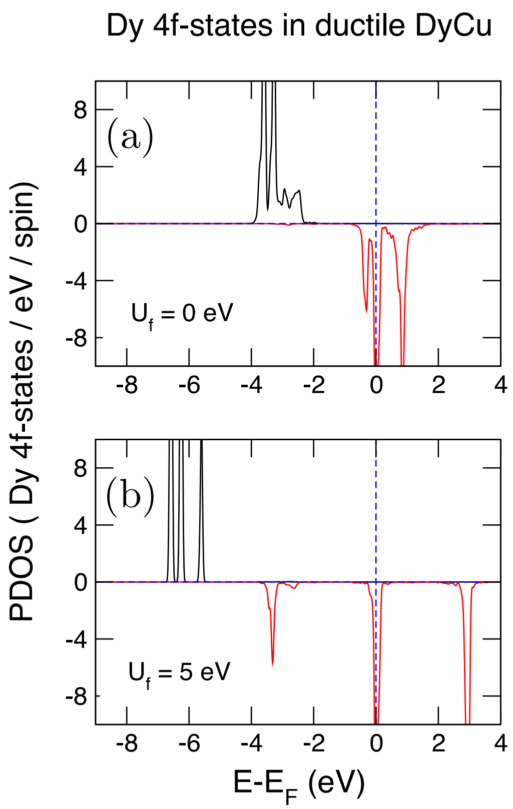

Supplementary Figure 3 | (Color online) **Dy-atom $4f$-orbital-PDOS in ductile DyCu system.** (a) Without the inclusion of Hubbard-U_f term. (b) With U_f = 5 eV. Ferromagnetic spin order imposed. Black and Red color represent spin-up and spin-down configurations, respectively. Blue dotted line at 0 eV indicate the position of the Fermi level (E_F). Data shown for DFT-PBE calculations using projector augmented-wave pseudopotentials.

Gschneidner *et al.* We anticipate the partially filled $4f$-electronic states of R-elements to play a predominant role in the magnetic and transport properties of these B2 intermetallics and perhaps not critical for the mechanical properties (an interpretation that is consistent with the calculations of Gschneidner *et al*). It is also interesting to note that our decision tree algorithm did not pick any of the orbital radii associated with the R-element for classification; although caution must be exercised, because these orbital radii are not readily suited for real electronic structure calculations. Clearly, more experimental works and accurate theoretical calculations are necessary to shed light on the impact of partially filled $4f$-states on the mechanical properties of these materials. For instance, in our data set (taken from Gschneidner *et al*[1]) only for (M=) Cu, Ag, and Au-based systems rigorous evaluation of mechanical properties for different R elements are reported. As a result, we find that our classification models are not sensitive to R-element.

SUPPLEMENTARY NOTE 4

MACHINE LEARNING

For the decision trees as implemented in `Weka`, we utilized the confidence factor value as 0.25 and the minimum number of instances per leaf was set at 2 (default values). We used a total of eight datasets (four and four for AB and RM compounds, respectively) in this paper and they are attached separately in XLS format.

* txl@lanl.gov

[1] K. G. Jr., M. Ji, C. Wang, K. Ho, A. Russell, Y. Mudryk, A. Becker, and J. Larson, Acta Materialia **57**, 5876 (2009).
[2] M. Topsakal and R. Wentzcovitch, Computational Materials Science **95**, 263 (2014).
[3] A. Georges, G. Kotliar, W. Krauth, and M. J. Rozenberg, Rev. Mod. Phys. **68**, 13 (1996).
